# The Moderating Effects of Social Support in the Relationship between Problem Drinking and Depression of the elderly disabled

**DOI:** 10.1192/j.eurpsy.2022.2109

**Published:** 2022-09-01

**Authors:** M.S. Yoon, H. Yu, M. Yu

**Affiliations:** Jeonbuk National University, Social Welfare, Jeonju, Korea, Republic of

**Keywords:** Depression, elderly with disability, social support, problem drinking

## Abstract

**Introduction:**

In recent years, the elderly population in Korea has rapidly increased, and the proportion of the elderly over 65 years old was 14% as of 2017. In particular, as the elderly population increases, the number of elderly people with disabilities has increased by 16.3% over the past 7 years, from 30.3% in 2010 to 46.6% in 2017. Based on the dual view of the elderly and the disabled, it is necessary to pay attention to the problems of drinking and depression in these relatively marginalized groups.

**Objectives:**

This study aimed to investigate the moderating effect of social support between problem drinking and depression of the elderly with disability

**Methods:**

This study analyzed the 12^th^ wave(2017) KWPS(Korean Welfare Panel Study) and Disability Study which included 195 elderly with disability aged 60 over. Collected data were analyzed by SPSS 21.0 and STATA.

**Results:**

First, the elderly with disabilities were more likely to be depressed than the women, living without spouse, the less satisfied with health, the higher the problem drinking, the lower the social support. Second, disability factors didn’t show any influence on the depression of subject and subjective health satisfaction significantly related to the depression. Third, social support moderated the relationship between the problem drinking and depression. According to the Quantile regression analysis, in group with low social support, the more the problem drinking, the higher the depression.

**Conclusions:**

Social support in elderly with disability was a significant factor for problem drinking and depression. The implication and limitation of these findings are discussed

**Disclosure:**

No significant relationships.

